# Web 2.0 and Internet Social Networking: A New tool for Disaster Management? - Lessons from Taiwan

**DOI:** 10.1186/1472-6947-10-57

**Published:** 2010-10-06

**Authors:** Cheng-Min Huang, Edward Chan, Adnan A Hyder

**Affiliations:** 1 International Injury Research Unit, Department of International Health, Johns Hopkins Bloomberg School of Public Health, 615 North Wolfe Street, Suite E-8132, Baltimore, Maryland, 21205, USA

## Abstract

**Background:**

Internet social networking tools and the emerging web 2.0 technologies are providing a new way for web users and health workers in information sharing and knowledge dissemination. Based on the characters of immediate, two-way and large scale of impact, the internet social networking tools have been utilized as a solution in emergency response during disasters. This paper highlights the use of internet social networking in disaster emergency response and public health management of disasters by focusing on a case study of the typhoon Morakot disaster in Taiwan.

**Discussion:**

In the case of typhoon disaster in Taiwan, internet social networking and mobile technology were found to be helpful for community residents, professional emergency rescuers, and government agencies in gathering and disseminating real-time information, regarding volunteer recruitment and relief supplies allocation. We noted that if internet tools are to be integrated in the development of emergency response system, the accessibility, accuracy, validity, feasibility, privacy and the scalability of itself should be carefully considered especially in the effort of applying it in resource poor settings.

**Summary:**

This paper seeks to promote an internet-based emergency response system by integrating internet social networking and information communication technology into central government disaster management system. Web-based networking provides two-way communication which establishes a reliable and accessible tunnel for proximal and distal users in disaster preparedness and management.

## Background

Disaster response has always been a challenge during and after major disasters due to the impact of disaster itself, the number of organizations and individuals participating in the response[1] and the lack of rapid social networking to support immediate community response. Disaster, regardless of etiology, exceeds the ability of the local community to cope with the event and requires specialized resources from outside the area impacted[2-4]. In a large-scale destructive event, one of the greatest challenges to public health workers and rescuing teams is to have stable and accessible emergency communication systems[5,6]. However, little researches currently exist regarding the use of communication platforms and internet social networks for emergency response.

Emergency response during disasters is often complicated because communication becomes unavailable. The Chi-Chi earthquake in Taiwan and Hurricane Katrina in US have proven that current telephone, radio and television-based emergency response systems are not capable of meeting all of the community-wide information sharing and communication needs of residents and responders during major disasters[7,8]. After 9/11, Preece and Shneiderman *et al *proposed the concept of community response grids[9] which would allow authorities, residents, and responders to share information, communicate and coordinate activities via internet and mobile communication devices in response to a major disaster. Information technologies has the potential to provide higher capacity and effective communication mechanisms that can reach citizens and government officials simultaneously[10].

### Concepts and Definition

Internet social networking (ISN) or online social networking is the use of web based technologies to provide a virtual forum for internet users however diverse and afar to communicate and share ideas and information [11,12]. The term "Web 2.0" has been popularized since the Web 2.0 conference in 2004 hosted by O'Reilly Media and MediaLive[13]. The appearance of Web 2.0, and the availability of broadband networks have significantly enhanced the capabilities of the internet. Web 2.0 was described as "Web as Platform," - entrepreneurs, software developers, and end users have transformed the internet into a highly interactive medium accessible through microcomputers, smart devices like cell phones, PDAs, and mp3 players. Examples of Web 2.0 tools include search engines (*e.g*. Google and Bing), encyclopedias (*e.g*. Wikipedia), videos and photos sharing (*e.g*. Youtube and Flickr), blogs and social networking websites (*e.g*. Facebook, Twitter, and Plurk).

Microblogging (e.g. Twitter and Plurk sites) is a social networking tool defined as " -a form of blogging for users to send brief text updates (usually less than 200 characters) or micromedia such as photos or audio clips to be viewed by anyone or a specific group which can be chosen by the user [14]." Microbloggers post the message to subscribers, and they in turn can forward the information to others. Microblogging has created a two-way communication platform where dissemination of information is timely and vast [15]. Research has suggested that microblogging has power and potential in a scientific context for exchanging ideas, interests and information for a specific community[16-18].

In Taiwan, the most popular social network microblogging tools are Plurk and Twitter. The amount of global Plurk users coming from Taiwan has increased dramatically from 18.6% in April 2009 to 34.9% in July 2009[19]. Facebook, another popular networking site with 300 million users worldwide, added more than 88,000 new users in Taiwan alone in just one week in July 2009[20]. These new social networking sites, once thought of as fresh online toys for the young, are effective social and public health tools as was demonstrated during typhoon Morakot and its aftermath in August 2009.

This paper is aimed at describing the implications of internet social networking among large web-users during major disasters in order to establish an integrated internet-based emergency response system. Microblogging/Blogging and social networking sites are distributed, decentralized technologies. These sites have empowered the public to share experience and information during emergency and disaster response activities. The following case study provides a lesson for policy makers to consider in introducing technology to enable civil society to dialogue with the official government when facing major disasters.

## Discussion

### Case Study: applications of social networking in Taiwain Morakot typhoon disaster

During August 8^th ^to 10^th ^2009, typhoon Morakot, a medium scale tropical cyclone, wrought catastrophic damage in Taiwan, affecting a large portion of Southern Taiwan and leaving over 600 people dead, 76 missing and 24,950 people displaced[21]. Typhoon Morakot ruined more bridges and roads than the disastrous earthquake of September 21, 1999[22,23]. The accumulated rainfalls in parts of southern Taiwan reached 2866 mm, breaking the Central Bureau of Weather's historic record[24]. This storm was the deadliest typhoon to impact Taiwan in the last 50 years and even now the economic loss is difficult to estimate.

The night of August 8^th^, typhoon Morakot caused historical record levels of rainfall in Southern Taiwan. Web users began reporting the real-time situation on the forum PTT http://pttemergency.pixnet.net/blog, one of the most popular internet social networks in Taiwan. PTT is a bulletin board system with more than 1.2 million registered users and an average 10,000 users online simultaneously. On August 9^th^, an unofficial Morakot Online Disaster Report Center was established by a group of internet users from the Association of Digital Culture Taiwan http://typhoon.adct.org.tw/. They advised fellow internet users living near areas battered by the storms to gather information, such as sustained damage or assistance needed on popular social networking websites including Twitter http://twitter.com/TaiwanFloods or Plurk http://www.plurk.com/floods. This website was then integrated into local governments' communication systems on August 10 and updated from the official disaster response center. Some users hosted Google maps on which residents who were waiting for rescuing could overlay information such as their current location and, the latest situation of damage caused by severe rainfall and landslide http://www.google.com.tw/intl/zh-TW/landing/morakot/. Plurk and Twitter users also sent messages to help rescuers acquire accurate position for their family and friends who live in affected areas.

In the initial stage of this typhoon disaster, major response operations were not coordinated efficiently. During the most crucial first few hours after the catastrophe, the Central Response Center underestimated the early scope and gravity of the disaster due to the lack of information and communication from affected areas. Official government communication early in the crisis failed, causing people to turn to websites run by non-governmental organizations, local media and individuals for information. For instance, when traditional emergency reporting systems in Tainan County were overloaded, people instead reported the first aid need directly on the Tainan Commissioner's Plurk. By using microblogging to compile data, local emergency medical system workers had successfully rescued 14 trapped people by the second day of typhoon Morakot[20].

In addition to these examples, Web 2.0 social networking also serves as a platform in resource gathering, logistics allocation and the distribution of relief supplies. Volunteering was another activity promoted through social networking services observed during typhoon Morakot. The social networking services were used to spread the news on volunteering opportunities and to match the users and volunteers to the time and location of need http://morakot.yam.com/.

### The application of ISN on Disaster Management

The goals/tasks of disaster management conducted by public health workers include:

1. Preventing unnecessary morbidity, mortality and economic loss resulting directly from a disaster. 2. Mitigating morbidity, mortality and economic loss due to the mismanagement of disaster relief efforts[25]. Therefore, the first priority is to understand the nature of disasters, and through this understanding, we identify the public health problems. Thus, collection, interpretation and dissemination of accurate and timely data from affected areas become necessary during and after the major disasters.

For public health workers and emergency responders, one of the most significant benefits of ISN is the speed to construct a network of professionals around practical, realistic common interests and objectives rather than around traditional bureaucratic structures. The non-hierarchical two-way communication system provided by most Web 2.0 social networks also empowers public users to participate in policy discussions with feedback to influence policy making.

The capacity of current emergency telephone and official communication system in Taiwan was drastically insufficient during the Morakot disaster. For example, the government was unaware of hundreds of survivors in mountainous areas of Kaohsiung county until informed by the news media. The primary reason that the information systems failed is not simply due to the difficult environment that the disaster created or its own internal limitations but rather, inadequate organizational capacity associated with a lack of support and oversight. The 911 emergency telephone system is not designed for disasters which resulted in difficulty in prioritizing or triaging the thousands of incoming calls. A good emergency communication system should be trustworthy, scalable, dependable, and reliable. When people are faced with uncertainty and lacking full knowledge of the risk, they will look to trusted sources of information for guidance[26].

Community response grids and microblogging can help reduce the gaps between residents and professional emergency rescuers in providing direct information during emergencies and understanding the severity and breadth of major disasters[9]. The technologies provide an online environment for information sharing and tracking. Used properly, microblogging can be a valuable component in an overall communication strategy. It is important to note that Twitter and other similar tools run on two-way communication which helps victims and professional emergency responders build relationships and share timely and important information directly with those in immediate need.

### Challenges to the Application and Use of ISN Tools

There are limitations and potential mishap of using ISN as a tool for disaster response[27]. First, in general, remote and less developed areas have more challenges in accessing the internet. It is also a reality that the less affluent and the less educated people have less access to information technology. Unfortunately, those residents are usually the most vulnerable. Second, there is the inherent potential problem underlying technologies themselves. Internet, Web 2.0, cabling, routers, networking, electricity grid etc all rely on good functional infrastructure to be effective, but disasters usually destroy infrastructure and interrupt the services. Third, how do we authenticate, validate, and ensure the accuracy of the messages in times of crisis and chaos? With free flow of information, it is very difficult to census and monitor social networking sites. Fourth, we should consider the scalability of those social networking sites. If they are not capable of handling the workload, they would only have a negative impact on efficiency and information sharing. Fifth, research has shown that social networking sites are not secured and private and personal information can be leaked to others[28]. Sixth, internet social networking alone is not a silver bullet in disaster preparations and relief. We must be prudent to integrate all appropriate technologies. During the disaster, lives were saved through the use of Geographic Information System (Google Map) and information was related to the rescue teams through the use of social networking. http://www.google.com.tw/intl/zh-TW/landing/morakot/. If the systems are implemented correctly, the government should consider integrating internet-based disaster response systems into emergency communication strategies as internet is becoming more widely used for information of all types.

A critical limitation of the approach is the fact that the application of ISN in crisis management around the world is still far from being realized. In low- and middle income-countries, the most significant obstacle impeding widespread internet usage is the widening gap between people with no access and unlimited access to ICT. Illiteracy, limited education, poverty, lack of local language websites and basic computer skills are some of the major factors that restrict the use of ICT by the general population[29]. Perception around age appropriate use may also hinder willingness to use ISN because Web 2.0 tools are often considered to be a recreational activity for young people. Using existing resources to develop a flexible ISN platform tailored to user's socioeconomic status and educational level would be ideal. Other challenges to the use of ISN in disaster management include concerns of technological, social and financial sustainability[30]. There is an increasing need for political support in order to provide an ISN framework to develop a disaster response system at different levels of government. The system's capacity to collect and use information will require human resources in order to truly strengthen humanitarian responses. We suggest that local government in districts and cities be empowered to develop and provide such a system.

We still know very little about how to measure the real impact of ISN and web 2.0 tools in emergency-related activity. So far, there have been very few analyses and no clear methodology exists for such an evaluation. While there is great interest in this topic, few measures of effectiveness have been developed. Nonetheless, we expect to see improvements in early warning of disaster response as a result of increasing ISN use.

## Summary

The example of typhoon Morakot in Taiwan had shown that the internet-based Web 2.0 platform, mobile communication technologies and social networking could alter interactions between government and communities they serve for positive benefit during disasters. During the period of typhoon Morakot, government agencies were far behind of news organizations and NGOs in understanding the extent of the damage and identifying where refugees were trapped after the storm. An internet-based emergency response system would have allowed people to use mobile telecommunications devices or wireless computers to report such occurrences to the government to facilitate search and rescue. By using telecommunications technology such as Web 2.0, microblogging and other ISN technologies governments could revolutionize infrastructure to help individual and communities respond to and recover from disasters. Ultimately, it can lead to policy decisions to develop and foster the use of internet-based emergency response systems. This can bring significant benefits to governments, communities, responders and residents faced with threats of natural disasters. By harnessing the power and presence of internet technology and of social networks, we can change the way in which responders and residents are able to share information about and deal with crises.

## Abbreviations

ICT: information communication technology; ISN: internet social networking

## Competing interests

The authors declare that they have no competing interests.

## Authors' contributions

All authors contributed equally on developing the concept described in this paper and read and approved the final manuscript.

## Pre-publication history

The pre-publication history for this paper can be accessed here:

http://www.biomedcentral.com/1472-6947/10/57/prepub
